# Views on primary prevention of cardiovascular disease - an interview study with Swedish GPs

**DOI:** 10.1186/1471-2296-11-44

**Published:** 2010-06-02

**Authors:** Louise Silwer, Rolf Wahlström, Cecilia Stålsby Lundborg

**Affiliations:** 1 Nordic School of Public Health, Göteborg, Sweden; 2 Halmstad University, School of Social and Health Sciences, Halmstad, Sweden; 3 Div of Global Health (IHCAR), Dep of Public Health Sciences, Karolinska Institutet, Stockholm, Sweden; 4 Family Medicine and Clinical Epidemiology, Dep Public Health and Caring Sciences, Uppsala University, Uppsala, Sweden

## Abstract

**Background:**

General practitioners (GPs) have gradually become more involved in the prevention of cardiovascular disease (CVD), both through more frequent prescribing of pharmaceuticals and by giving advice regarding lifestyle factors. Most general practitioners are now faced with decisions about pharmaceutical or non-pharmaceutical treatment for primary prevention every day. The aim of this study was to explore, structure and describe the views on primary prevention of cardiovascular disease in clinical practice among Swedish GPs.

**Methods:**

Individual interviews were conducted with 21 GPs in southern Sweden. The interview transcripts were analysed using a qualitative approach, inspired by phenomenography.

**Results:**

Two main categories of description emerged during the analysis. One was the degree of reliance on research data regarding the predictability of real risk and the opportunities for primary prevention of CVD. The other was the allocation of responsibility between the patient and the doctor. The GPs showed different views, from being convinced of an actual and predictable risk for the individual to strongly doubting it; from relying firmly on protection from disease by pharmaceutical treatment to strongly questioning its effectiveness in individual cases; and from reliance on prevention of disease by non-pharmaceutical interventions to a total lack of reliance on such measures.

**Conclusions:**

The GPs' different views, regarding the rationale for and practical management of primary prevention of CVD, can be interpreted as a reflection of the complexity of patient counselling in primary prevention in clinical practice. The findings have implications for development and implementation of standard treatment guidelines, regarding long-time primary preventive treatment.

## Background

During the past few decades, there has been a substantial increase in prescribing of medicines for prevention of cardiovascular disease (CVD) in Sweden as well as in other countries [1,2]. The former reluctance to prescribe for example lipid-modifying agents [3] seems to have been overcome in Sweden, although internationally they are still considered to be underused [4,5]. More and more attention has also been paid to research on the influence of lifestyle factors, such as smoking, stress, alcohol consumption, exercise and food habits, on long-term cardiovascular health [6-8].

Most doctors in primary care are faced with decisions about pharmaceutical or non-pharmaceutical treatment for primary prevention everyday, i.e., decisions regarding treatment of individuals with risk factors but without manifest disease. There are ethical consequences involved, since treatment may lead to perceptions of illness when the 'healthy' patient becomes medicalized [9,10]. Information on indicators of risk for cardiovascular events, and the risks of lifestyle factors is frequently featured in the mass media, and public awareness has been considered high in Sweden [11]. During recent years, different models have been introduced for calculating and scoring risk as aids to treatment decisions [12]. Risk interpretation and risk communication are important topics for debate and research [13].

According to the Swedish definition of hypercholesterolemia (>5 mmol/l serum), 90-95% of the population between 50-70 years of age are hypercholesterolemic. One quarter of the population, from 20 years of age, is defined as hypertensive [6,14]. According to an analysis from 2005, based on individual data from a new Swedish health database, 53% of the Swedish population aged 60 years or more purchased prescribed preventive cardiovascular drugs, such as antihypertensive or serum-lipid reducing agents [15]. Guidelines have been changed from time to time and when the standard levels are lowered, an increasing fraction of the population is likely to be exposed to preventive actions, leading to more frequent situations involving information and treatment decisions. The primary level of health care in Sweden is organized in health centres with specialists in general practice, hereafter called general practitioners (GPs). GPs have a key role in drug prescribing and to provide information and make decisions regarding preventive actions in relation to cardiovascular disease. Previous studies have shown that views on treatment options can differ greatly also within a seemingly homogenous group such as Swedish GPs [3,16]. Awareness of such differences in views may also influence how guidelines are actually translated into clinical practice and are therefore of interest for development of treatment guidelines, as well as for GPs' reflections and discussions in their everyday practice.

The aim of this study was to explore, structure and describe the views on primary prevention of CVD and the experiences in clinical practice among Swedish GPs.

## Methods

In order to be able to explore views in an unlimited way, a qualitative approach was applied. The analysis was inspired by phenomenography, that aims to investigate the different ways in which people understand a particular phenomenon and present the variation in the form of categories of description [17,18].

Twenty-one GPs in Halland, a county in the South of Sweden, were interviewed and the interviews were analysed qualitatively to explore and structure the spectrum of views on primary prevention of CVD. Purposive sampling was used in order to ensure participation of GPs of different age, gender, length of experience, type of health centre (public or private) and recruitment area (urban or rural). The twenty-one doctors, 13 male and 8 female (Table 1), were recruited step-wise from a total of 50 GPs. First, the GPs were contacted by a preparing letter and asked to confirm their willingness to participate in the study by e-mail or telephone. They were informed of the voluntariness and they were also informed about their right to anonymity. Those who did not respond the first time were contacted again by e-mail or telephone. When a GP denied participation, a GP with similar characteristics was contacted. The most common obstacles to participation were a heavy workload and lack of time. All GPs included in the study were working in the same county council area, using the same treatment guidelines.

**Table 1:** GP characteristics

Gender:	Female	8
	Male	13
Age:	<50 years	8
	>50 years	13

Education:	Full, specialist	16
	Specialist training	5

Native language:	Swedish	17
	Other	4

Type of Health centre:	Public	16
	Private	5

Patient recruitment area:	Urban	10
	Rural	11

All 21 GPs were interviewed by the first author (LS) and all interviews were audio-tape recorded and lasted 30 to 60 minutes. They were conducted during the period October 2004 to March 2005. Informed consent was signed before the interviews started. Sixteen of the interviews were made face to face, and five were telephone interviews. These five GPs declined participation in a face-to-face interview, due to perceived lack of time, but accepted a telephone interview. They were included as they had unique combinations of characteristics (Table 1). The interviewees were encouraged to describe and reflect upon examples from their own practice. The emphasis was placed on the doctors' personal views and experiences, and not on their knowledge or perceptions of national or regional guidelines. The interviews were semi-structured and followed an interview-guide with suggested questions and probing areas (Table 2). Depending on the flow in the interview, and the expressiveness of the interviewee, the interviewer asked the questions more in general or in detail but aimed at having the main topics covered. First the topic was introduced in a general way, and if necessary, probing questions were asked to ensure that the expressed views covered the topics.

**Table 2:** Interview guide

Main topics	Probing questions
Communication of risk and risk reduction	How do you usually communicate/inform the patient of the results of the tests and of risk - numerically, graphically or how? How do you explain what it means?
	Most important to be said?
	"Typical" patient reaction?
	Numerical or non-numerical presentation of benefits and risks? Always talk about non-pharmaceutical treatment?
	Risk for you not to prescribe - in hypertension/in hyperlipidemia?
	Talk about follow-up, treatment targets, treatment length and possible adverse effects?

Treatment decision	How is the treatment decision made - what is the role of the patient? What would the optimal situation be?
	How often pharmaceuticals immediately?
	Difference in handling of hyperlipidemia and hypertension? Difference in handling of patients of different ages?

Other aspects of primary prevention	Has there been any shift in your view of primary prevention in later years - why?
	What is the most difficult about primary prevention of CVD?
	How would you treat yourself and your personal risk factors?
	Is there anything you would like to add regarding primary prevention?

The audio-tape recorded interviews were transcribed verbatim by the first author (LS) and the transcripts were checked by re-listening. All transcripts were read several times and expressions related to the aim of the study were marked in the texts. The first author (LS, a pharmacist) conducted the initial analysis, while the two other authors (RW, a specialist in family medicine, and CSL, a pharmacist), both with long experience of qualitative analysis of interview material, participated as co-evaluators. To get an overview, and facilitate the analysis, the marked expressions were transferred to an Excel-file with one column for each of the 21 interviews. The expressed views were coded and through further analysis of the meaning of each coded expression, two main categories gradually emerged. Expressions related to these were then divided into separate files for further structuring, analysis and description of the variation in views within the main categories and sub-categories. During the whole process, the material was related to the full transcripts, not to lose the original context. The authors carried out a continuously cooperative analysis resulting in a structured presentation in main categories and sub-categories of the variation in views on primary prevention of CVD among GPs.

The study was approved by The Regional Ethical Review Board in Göteborg, Sweden, registration number 241-04. Participation was voluntary and the participants were informed that they could withdraw from the study at any time. They were guaranteed confidentiality according to existing rules.

## Results

During the analysis of the interviews, two main categories of description emerged:

i) Degree of *reliance*, as a comprehensive term for belief in research data regarding the predictability of actual risk of cardiovascular disease, and regarding the possibilities or ways to prevent disease by primary prevention;

ii) Allocation of *responsibility*, regarding different aspects of primary prevention.

For each of these main categories, the variations in views in the different sub-categories are presented in the text, and summarised in table 3. A distinctive variation was found in the ways GPs experienced management of people with cardiovascular risk factors and in their beliefs in primary prevention of CVD. Some had, according to their own statements, changed from emphasizing pharmaceutical treatment to non-pharmaceutical measures (here defined as miscellaneous lifestyle changes), while others had changed in the opposite direction during the same period of time. No obvious pattern of linked views was found, but rather different combinations of independent views. Some doctors shared their experiences of the complexity regarding both reliance and responsibility.

**Table 3:** Summary table

RELIANCE	*Fields of agreement*	
	Too difficult and inappropriate to give the patient a numerical estimate of cardiovascular risk or risk score; more important to treat hypertension than hyperlipidaemia pharmaceutically; lifestyle intervention is more important for younger people; reduced drug prices influence prescribing criteria; individualization of advice and treatment is necessary; testing and prescribing drugs without proper indication can make patients feel ill	

	***Fields of variation***	

	1. Trust in pharmaceutical prevention	A. Firm trust in effectiveness and cost-effectiveness of pharmaceutical prevention
		B. Some doubts about the effectiveness for the individual patient
		C. Expressed doubts due to insufficient evidence

	2. Trust in non-pharmaceutical prevention	A. Non-pharmaceutical treatment is the basis
		B. Non-pharmaceutical treatment is too difficult to carry out and not very effective
		C. Non-pharmaceutical treatment is not effective and it impairs the quality of life for the patients

	3. Importance of a treatment goal	A. A pre-defined target is necessary
		B. The importance of a target is varying: important for high-risk patients but not for low-risk patients
		C. The lowering of blood-pressure or lipids is the most important, not to a certain value

	4. Pharmaceutical prevention for different ages	A. More important for younger individuals
		B. Equally important for young and old
		C. More important for older individuals

**RESPONSIBILITY**	***Fields of agreement***	

	Genetic disposition is a significant risk factor; the "western" lifestyle causes cardiovascular disease; smoking is the most important lifestyle risk factor; a positive expectation that the intervention is beneficial is a necessary condition for compliance	

	***Fields of variation***	

	1. Information regarding potential adverse effects and length of treatment	A. Important to inform for the patient to be prepared and feel safe
		B. Avoiding information is better, not to worry the patient

	2. Treatment decision	A. The doctor has the main responsibility
		B. A decision on equal terms is preferred
		C. The patient has the main responsibility

	3. The role of the doctor in non-pharmaceutical treatment	A. The doctor's role is to confront the patient with uncomfortable recommendations and make demands on the patient
		B. The doctor's role is to communicate a positive message and encourage every little step the patient takes towards a healthier lifestyle
		C. It is too difficult for the doctor to talk about lifestyle risk factors without blaming or burdening the patient. The doctor has no important role in non-pharmaceutical treatment
		D. The doctor has no right to demand a change in the patient's lifestyle, but should instead prescribe pharmaceuticals for smokers and over-weight people

*"In one way we medicalize an unhealthy lifestyle, we give medicines as a substitute for people taking care of themselves... but it is not that simple either to do without (medicines), because if you do, things can happen..." *(GP18)

*"I control the information, the prescribing decision is shared, but whether or not they then purchase and take the medicines, I don't control that..." *(GP16)

### Reliance

There were some recurrent topics regarding reliance, where quite uniform views were expressed: that it is often inappropriate or too difficult to present the patient with a numerical estimate of risk or risk score; that it is more important to treat hypertension than hyperlipidaemia pharmaceutically; that lifestyle intervention, such as smoking cessation, is more important for younger people; that it is necessary to individualize the advice and treatment given; and that there is a risk of making patients feel ill by ordering tests without a proper indication, and by prescribing drugs.

The belief in an actual predictable risk for the individual, and the reliance in effectiveness of primary prevention of CVD varied, which will be described in the following four sub-categories with the internal variation illustrated by expressions:

1) Practical experience of actual risk of CVD, and trust in pharmaceutical prevention of CVD by lipid-lowering or antihypertensive treatment:

A: Firm trust in the scientific documentation of effectiveness for the individual and of cut-off points as true levels of increased predictable risk. Pharmaceutical treatment is cost-effective and saves money by reducing the number of myocardial infarctions and stroke, and thereby the related intensive care.

*"... it must be the medicines that did it, mustn't it, it saved lots of money, I think, it's costly intensive care, MI and stroke and those things" *(GP 2)

B: Some doubts about the effectiveness for the individual, but acceptance of the guidelines as rules to obey (even if they change over time), hoping and wishing that one is doing the best for the patient. Primary prevention was considered time-consuming and more difficult than secondary prevention, as the evidence in its favour is not unambiguous.

*"How much should you treat people who feel well and are healthy, so to speak, and who have risk factors, I don't think that is completely obvious, it is not, and it would be a bit heavy if too much of your time would be consumed by treating prospective risks... In a way it's easier when there is a disease and you know that you are doing more good*. (GP 7)

C: Expressed doubts, due to the meagre evidence for long-term treatment, both in terms of beneficial and adverse effects. The pharmaceutical industry puts pressure on the prescribers. The benefit for the individual was considered to be marginal and the doctor's duty is among other things to tone down the risks and relate them to other risks in life.

*"...but there is a pharmaceutical industry that puts pressure on us, it's in newspapers etc, we are continually fed with this... and I think it is as much my duty to sit here and tone down the risks for the young ones, above all. It doesn't seem reasonable that the majority of the population should take medicines" *(GP 10).

2) Trust in non-pharmaceutical prevention:

A: Non-pharmaceutical treatment is the basis, with the greatest chances and the least adverse effects, while pharmaceutical treatment is a supplement. The project "Physical activity on prescription" [19] was mentioned as a positive example.

*"And the lifestyle is like the main thread in the whole treatment, getting tablets doesn't mean that you don't have to change your lifestyle" *(GP 13).

B: Doubts about the effectiveness of non-pharmaceutical treatment as it is difficult to carry out. Most patients are elderly and for them it is even harder to be physically active or to change their eating habits in a radical way and then it is better to prescribe pharmaceuticals.

*"Because most patients you see in real life are elderly, and there you only find high levels, and you realise that you can give this advice about their lifestyle, but they will not be very effective on this person so you'd better prescribe pharmaceuticals" *(GP 1)

C: Non-pharmaceutical treatment is not effective and it is important, in primary prevention, to avoid negative impacts on quality of life through changes in lifestyle, since we are mostly dealing with people who feel healthy before they get treatment.

*"...and I must say that sometimes I imagine myself in the choice between changing my eating habits... and remembering and thinking a lot about what I eat... and taking a tablet ... I would prefer taking a tablet and go on living as usual..." *(GP 9)

3) Aiming at a numerical value of treatment target or not (for blood pressure and blood lipids):

A: Awareness of a target for the treatment is a necessary condition to enable the patient to see that the treatment is effective.

*"...We define what we aim at when we start the treatment. The patient should always know what target he or she should reach" *(GP 13).

B: The importance of a numerical treatment target varies in each specific case. For high-risk patients, defined goals are important, but for low-risk patients they are not. For some GPs, the importance of a defined target was mainly related to hypertension, but regarding hyperlipidemia it was not considered so important.

*"...if there is a high risk, you set clear goals, if there is a fairly low risk, I don't talk very much about it but start treatment ...I think you have more defined goals when you treat blood pressure..." *(GP 9).

C: Lowering the blood pressure or blood lipids in general is the most important thing, rather than aiming for a certain value, as long as the evidence is too poor to clearly define treatment targets.

*"...and I think it is reasonable that as long as we do not know, we should not just look at target values" *[regarding lipid-lowering] (GP 15).

4) Consequences of age regarding pharmaceutical treatment (younger = 40 years; older = 70 years):

A: It is more important to treat younger people, as they are likely to have more years to live.

*"Yes, you drill the 40 year-old much more. But the 70 year-old will comply better... yes, you have a completely different ambition for the 40 year-old... and you start pharmaceutical treatment faster on the 40 year-old" *(GP 18).

B: It is equally important to treat younger and older people, while on the one hand a cardiovascular event is more likely during the next 20 years for the older person, and on the other hand, the probability of getting CVD before arriving at a very old age is greater for the younger one.

*"Looking at the arguments from two different positions you might consider it important in both cases" *(GP 19)

C: Treating older people is more important, because knowledge is lacking about the effectiveness of starting a treatment at a young age which is intended to continue for 30-40 years.

*"...the risk during the following years is much higher if you are older... and most of what we know about is just how to prevent events during the first few years ahead. How much you prevent atherosclerosis by treating a 25 year-old, to prevent a possible infarction at the age of 50 or 60... we know very little about that ... it takes higher levels for younger than for older people" *(GP 9)

### Responsibility

The significance of genetic disposition as a risk factor was frequently emphasized, and regarding the responsibility for avoidable risk factors, the "western" life style was uniformly seen as a cause of cardiovascular disease, both at population level and individual level. Smoking was seen as the most important lifestyle risk factor. A quite general view was also found regarding responsibility for compliance and outcome of pharmaceutical treatment. A firm conviction, that the intervention is beneficial, was considered a fundamental prerequisite for patients being able to take their medicine regularly, even if they feel healthy. Here, the doctor's persuasive attitude towards the patient, creating a positive expectation, was considered important.

However, in three sub-categories there was a variation in views on responsibility regarding different aspects of primary prevention:

1) Information of potential adverse effects of pharmaceuticals and the likely length of treatment:

A: It is important to inform about potential adverse effects of pharmaceuticals or probable length of treatment from the beginning, for the patient to be prepared and feel safe.

*"I always inform the patient about adverse effects initially and then they report if they get any" *(GP 13).

B: It is better to avoid informing the patient about adverse effects or the length of the treatment at the outset, in order not to worry the patient.

*"You never talk about the eventuality of life-long treatment, never during the first meeting... I think that is like shoving it down their throats when they feel quite weak already... that's bad timing" *(GP11).

2) Shared responsibility (doctor and patient) for the treatment decision:

A: The doctor has the main responsibility, because he or she has the adequate skills and enjoys the patient's confidence to make the decisions, and because the patients sometimes make themselves dependent and are unwilling to decide.

*"...I don't understand how they could possibly make their own assessment - in most cases they don't have any basis for assessment or decision for that, so to speak" *(GP 3)

B: A decision on equal terms is preferred, as the doctor has knowledge about the disease and the patient about his or her personal situation. The discussion between the two of them is important, and they usually arrive at an agreement.

*"...yes it's on equal terms, I have the knowledge of the disease and the patient of himself or herself and their personal situation, and we try to reach a good conclusion from that" *(GP 20)

C: The patient has the main responsibility, and this is a prerequisite for compliance. This category also includes the views that in order to motivate the patient, the doctor could inform (or persuade) the patient, but the decision is the patient's alone.

*"...When I feel that they have understood, I can relax, whereas they can make the decisions regarding their own lives" *(GP 9)

3) The doctor's role and responsibility in non-pharmaceutical treatment:

A: The doctor's role is to confront the patient with uncomfortable recommendations regarding the patient's own responsibilities, as it is necessary for a successful pharmaceutical treatment to demand that the patient carries out non-pharmaceutical activities.

*"If they smoke a lot, I usually tell them that it would be ridiculous to treat your blood pressure and your cholesterol if you keep on smoking. There is no point in doing that. I am usually quite mean then" *(GP 4)

B: The doctor's role is to communicate a positive message about what one can do oneself, in conjunction with the primary diagnosis of an elevated blood pressure or lipid level. This role is also important in making the patient ponder over what they might do themselves and what interventions might be realistic in their situation, and further, in encouraging every little step the patient takes towards a healthier lifestyle.

*"You want somehow to give them something positive to cling to... that if I can do this and that and I can stop smoking or I can go down in weight or if I can be a little more physically active, I will have lots to gain" *(GP 1).

C: It is difficult to talk about the risk factors in the patients' lifestyle, without blaming or burdening them. The doctor's role in lifestyle change is insignificant and the roles of nurses and dieticians are more suited for such communication in health information talks and follow-ups, but there are too few dieticians.

*"As a doctor you can do very little to help directly regarding overweight... and furthermore it's a topic covered with shame..." *(GP 9)

D: The doctor has no right to demand a change in the patient's lifestyle which makes pharmaceutical treatment even more important to smokers and over-weight people.

*"I don't consider myself having the right to demand that people stop smoking. I think it is presumptuous to make such strong demands." *(GP 11)

## Discussion

The views on different aspects of primary prevention varied, despite the fact that all the GPs in our study worked in the same county and were recommended to use the same guidelines which theoretically could have restricted the variation. There could have been a risk that GPs that were especially interested in pharmaceutical treatments or up-to-date regarding guidelines would be more willing to participate in the study and share their time to discuss these subjects, but such a selection is unlikely as a wide variation in views was found. The combination of participants in the study, together with the fact that the local guidelines were very similar to the national guidelines and also to the European guidelines, could be considered a ground for transferability of the results, as these local experiences might be assumed to be universal and to reflect those of GPs in other parts of the country, and probably also in other countries.

By asking the GPs to present their own descriptions of managing cases of primary prevention, material that was close to their actual practice was received and could thereby be considered as reflecting their genuine views. The credibility of this study is also strengthened by the fact that all interviews were performed by one interviewer, although the fact that the interviewer was known to be a pharmacist, at the time employed by the National Corporation of Pharmacies in Sweden, might have affected the dialogue on pharmaceutical treatment to some extent. Five of the interviews were made by telephone, to save time and enable selected GPs to participate. These interviews were however as comprehensive as the face-to face interviews and were therefore included in the material.

The credibility of the analysis of the study is enhanced by the way the three authors' analysed and continuously discussed the interview material. Two of the authors have long experience in analysing this type of qualitative material, in particular interviews with doctors [3,16].

The two main categories, degree of reliance and allocation of responsibility, were interlaced, with responsibility appearing as a consequence of reliance in terms of opportunities to prevent disease. The view of cardiovascular risk, as an actual risk for the individual, could be seen as a central condition for the belief in a capability to reduce risk in any way. Using numerical risk or numerical risk reduction, in communication with the patient, was mostly considered inappropriate or too difficult.

Some GPs in our study felt a great responsibility to treat the patient in an optimal way, pharmaceutically or non-pharmaceutically, based on their knowledge of research data on numerically reduced risk. They were anxious about not doing well enough as if they were in a way having the patients' lives in their hands, already in the case of primary prevention. Others considered that in order to act beneficially towards the patient, their informative role and responsibility was to relate the magnitude of CVD risk to other risks in the patients' lives. Good risk communication is often considered a prerequisite for patient involvement in treatment decisions and GPs are supposed to have an important role in communicating the meaning of risk [13]. A tendency to overestimate absolute cardiovascular risk has been reported for both physicians and patients [13] and in other studies the physicians were found to usually tell the patient that an adverse outcome was certain, unless the patient adopted the recommended treatment [20,21]. It has also been stated that patients were less likely to take antihypertensive drugs if they had accurate information about their levels of risk [22]. An interpretation could be that compliance by asymptomatic individuals, to take pharmaceuticals regularly for decades, is correlated to fear of disease or death. The risk of making healthy individuals feel ill through primary prevention, as expressed by some GPs in our study, therefore seems impossible to avoid.

Although the respondents uniformly put forward genetic disposition as the background for disease, they also expressed thoughts about the individual's responsibility for risk factors. Some of the GPs thought that pharmaceutical treatment was considered more important to smoking and overweight individuals, as they felt that the doctor has no right to demand a change in the patient's lifestyle. Also in some other studies, the fear of moral intrusion or placing guilt on the patient has been expressed [23,24]. Preventive thinking has been said to be founded in part on perceptions of causal connections that assign responsibility to the individual [9], but it has also been stated that great confidence in modern medical science, could impede changes in life style [25].

We found contradicting views regarding the reliance on research data in relation to the effectiveness of prevention. The ideas expressed in the sub-categories varied crosswise in different dimensions and did not appear as linked, since for example reliance on effectiveness of pharmaceutical prevention did not exclude reliance on the effectiveness of non-pharmaceutical prevention. In a British interview study from 1994, many findings were similar to ours: that the profession creates fear and a creeping medicalization in the population, that the lack of evidence for risk identification and interventions is problematic, and that interpretation of probabilistic epidemiological data is difficult in the individual case [23]. Also in an American survey of practising family physicians, regarding treatment of hyperlipidemia, a large variability in beliefs and practice patterns was found [26].

Participants in our study expressed the view that the role of the physician is different in primary and secondary prevention. In a Scottish qualitative study from 1998, GPs were reported to be less clear about the evidence for statins in primary prevention than in secondary prevention, and found social and economic issues more complex regarding primary prevention [27]. The results of another qualitative British interview study from 2003 showed that the GPs experienced difficulties in interpreting primary prevention risk assessment tools, and they also had concerns about increased workload and medicalization [28]. In a French qualitative interview study, patients and physicians agreed on the difficulties associated with implementing lifestyle changes and adhering to long-term treatment [29].

The GPs in our study had various views on the role of the patient in the treatment decision, and they also expressed different views about the responsibility of the physician for the outcome of the drug treatment or the lifestyle intervention. In terms of treatment decisions, similar variations in views among physicians about patient participation were found in another study of Swedish GPs [16]. Other studies also report that time constraints inhibit information provision, and that there could be a risk that encouraging patients to choose between competing treatment options would place an additional burden on the patients, and lead to unnecessary anxiety and stress [23,24]. When patients are involved in treatment decisions, they have to realise the possible harms and benefits of the choices they face [30]. It has been stated in the literature, that preventive work is rendered even more difficult when combined with professional uncertainty, as in the case of pharmaceutical primary prevention [21]. In an interview study with Danish GPs, about their feelings of discomfort or pleasure regarding prescribing different groups of medicines, statins were mainly graded at the side of discomfort [31]. Preventive treatments, and the sick role thereby imposed on a great number of people, were among the most difficult areas and were put forward as problematic fields regarding Danish GPs' autonomy and self-perception [31,32].

The interviews were performed during the years 2004 to 2005, and guidelines regarding cardiovascular prevention have been revised since then - the national guidelines in 2006 and the European guidelines in 2007 [33,34]. However, it is unlikely that these new guidelines, although well developed, would change the deeper personal views and beliefs to any substantial extent. The summary of the ESC (European Society of Cardiology) guidelines also emphasizes, among other things, that "The preventive interventions must be based on a patient-centred approach, where the doctor pays full attention to appraise and meet the patient's concerns, beliefs, and values, and respects the patient's choice even if it is not in concordance with the doctor's first proposal. To change lifestyle or take medication often means for the rest of the patient's life, so the decision must be owned by the patient" [34]. Balancing the patient's beliefs and values, the national or international guidelines and the doctor's beliefs and values will still be a daily task for a GP. Our findings further emphasize the necessity of a continuous discussion among GPs, regarding managing primary prevention in clinical practice.

## Conclusions

Despite uniform guidelines, the views and beliefs about the beneficial effects of primary prevention of CVD varied considerably among the GPs, which might be interpreted as a reflection of the complexity of patient counselling in this field. The findings should be used to stimulate discussions regarding primary prevention of CVD among GPs, and could also add information when developing modified treatment guidelines, regarding long-term primary preventive treatment, and when planning for the implementation of such guidelines.

## Competing interests

The authors declare that they have no competing interests.

## Authors' contributions

All GPs were interviewed by the first author (LS) who also conducted the initial analysis, while RW and CSL worked as co-evaluators. LS wrote the drafts of the manuscript, which were commented for intellectual contribution by RW and CSL. All authors read and approved the final manuscript.

## Pre-publication history

The pre-publication history for this paper can be accessed here:

http://www.biomedcentral.com/1471-2296/11/44/prepub
